# Adverse events and in-hospital mortality: an analysis of all deaths in a Norwegian health trust during 2011

**DOI:** 10.1186/s12913-017-2417-7

**Published:** 2017-07-06

**Authors:** Hans Flaatten, Guttorm Brattebø, Bjørn Alme, Kjersti Berge, Jan H. Rosland, Asgaut Viste, Bjørn Bertelsen, Stig Harthug, Sidsel Aardal

**Affiliations:** 1 Department of Anaesthesia & Intensive Care, Haukeland University Hospital, Bergen, Norway; 2 Department of Clinical Medicine, University of Bergen, Bergen, Norway; 3 Department of Research and Development, Haukeland University Hospital, Bergen, Norway; 4 Department of Medicine, Haraldsplass Deaconess Hospital, Bergen, Norway; 5 Department of Pathology, Haukeland University Hospital, Bergen, Norway; 6 Department of Clinical Science, University of Bergen, Bergen, Norway

**Keywords:** Adverse events, Hospital, Death

## Abstract

**Background:**

The estimated number of in-hospitals deaths due to adverse events is often different when using data from deceased patients compared with that of a population experiencing adverse events.

**Methods:**

The study was conducted at three hospitals in the Bergen Hospital Trust, including a 950-bed university hospital. The objective was to study the reported deaths and investigate the probable number of deaths caused by adverse events. Information about all patients who died in the hospitals during 2011 was retrieved from the electronic patient data management system and the medical records. All deaths were classified into two groups according to Norwegian law based on whether or not the death was sudden and/or unexpected. The cause of death in the latter group was further classified as being due to either natural or unnatural causes according to national requirements. An expert review panel screened the patient records for information regarding adverse events and possible (≥ 50%) preventability. Age, length of hospital stay, and Charlson Comorbidity Index were also registered.

**Results:**

There were 59,605 unique patients admitted in 2011 and 1185 registered deaths (1.98%). The mean and median ages of the deceased were 73,8 and 78 years, respectively, and the median length of stay was 5.6 days (range). Of these deaths, 290 (24.5%) were considered sudden and/or unexpected and 218 were considered to be due to natural causes. Of the 72 unnatural deaths, 16 (1.4%) were classified as preventable or probably preventable. For 18 deaths (%) it was impossible to confirm or rule out preventability.

**Conclusions:**

Using this method, we identified a small proportion of hospital deaths that could be classified as unnatural. Furthermore, there was a ≥ 50% chance or more that 34 deaths (2.9%) were due to causes that could have been prevented.

## Background

Death is a common occurrence in hospitals, partly because of the increased number of patients with severe and complicated chronic end-stage diseases and partly as a consequence of the increased severity of the illness in many acutely admitted patients. Hospital deaths can also occur partially or totally as a consequence of adverse events that occur during medical care [1]. The magnitude of the latter group is debatable and depends on what technique is used to identify the events [2].

Recently, two studies from Europe were published that primarily studied cohorts of patients dying in hospitals with regards to the contribution of adverse events to these deaths. In the UK, 1000 deaths in 10 hospitals were retrospectively reviewed. Of these, 5.2% of the deaths were judged as preventable [3]. In a similar investigation from the Netherlands, 3983 admissions of patients who died in 21 hospitals were studied. In that study, 4.1% of deceased patients had a preventable adverse event prior to death [4]. To our knowledge, no similar cohort studies of deceased patients in which the occurrence of adverse events contributing to hospital deaths has been described has been performed in any of the Nordic countries.

The Bergen Hospital Trust, Norway, is composed of one large regional university hospital and two local hospitals. In order to better understand the occurrence of hospital deaths in general and, in particular, adverse events contributing to deaths in our hospitals, we analysed all hospital deaths that occurred in 2011. The primary aim was to describe the causes of death in all deceased patients according to Norwegian law and, in particular, the group of unexpected deaths that includes deaths related to treatment of disease or trauma (Table 1). We also wanted to further investigate those deaths that could be attributed to an adverse event and whether those deaths could have been prevented. Furthermore, we wished to describe the epidemiology of our hospital deaths with regards to clinical departments, times of death, age, gender, and co-morbidities of the patients who died.

**Table 1:** List of unnatural causes of death according to Norwegian law

Death is considered to be unnatural if it was caused by:
A. Murder or other trauma to the human body
B. Suicide or self-inflicted damage to the body
C Accidents including capsizing, burns, avalanche, lightning strikes, drowning, traffic-related incidents
D. Occupational accidents
E. Error, omission, or accidents related to diagnosis or treatment of disease or trauma
F. Use of illegal drugs
G. Unknown causes when death has occurred suddenly and unexpectedly
H. All deaths occurring in civil or military prisons
I. Finding of an unidentified corpse

This list is the official list of unnatural deaths in Norway (§ 2 Norwegian Regulations 2000–12-21 nr. 1378)

## Methods

Bergen Hospital Trust is the largest health trust in Helse Vest, the western-most of the four Norwegian health regions. The main hospital is Haukeland University Hospital, a 950-bed tertiary referral hospital serving 1.1 million inhabitants (approximately 20% of the Norwegian population). It is also the local hospital for the 350,000 inhabitants of Bergen, the second largest city in Norway. The hospital performs all types of medical services with the exception of organ transplantation and replantation surgeries. There is also a small community hospital (Voss) included in Bergen Hospital Trust and one private community hospital in the health trust (Haraldsplass Diaconal Hospital). Data from all three hospitals were included in the study.

During 2011, the hospitals treated 59,605 unique in-patients. In Norway we have no separate Emergency departments, only emergency rooms. Hence all patients are assigned to one of the hospital departments at admission. Patients were automatically registered according to defined criteria at discharge as alive or dead. The hospital records (medical files) for all deceased patients in our hospital trust in 2011 (eligible criteria), were retrieved from our electronic administrative and medical records system (DIPS-EPJ-Distribuert Informasjons og Pasientdatasystem i Sykehus, an application developed in Norway). The study size was as such predefined to this group, *n* = 1185 hospital deaths.

For all deaths, the patients’ age, gender, date and time (hour of the day) of death from the hospital admission, and the department responsible for the patient’s care at the time of death, were also registered. In addition, the Charlson Comorbidity Index [5] was calculated using the 10th revision of the International Statistical Classification of Diseases and Related Health Problems (ICD-10) admission primary and secondary diagnoses.

Because of the large amount of records to screen we used a two stage strategy: an initial screening of all death records, then an in-depth consensus discussion of the sudden and unexpected deaths identified in the first screening.

**Fig. 1:**
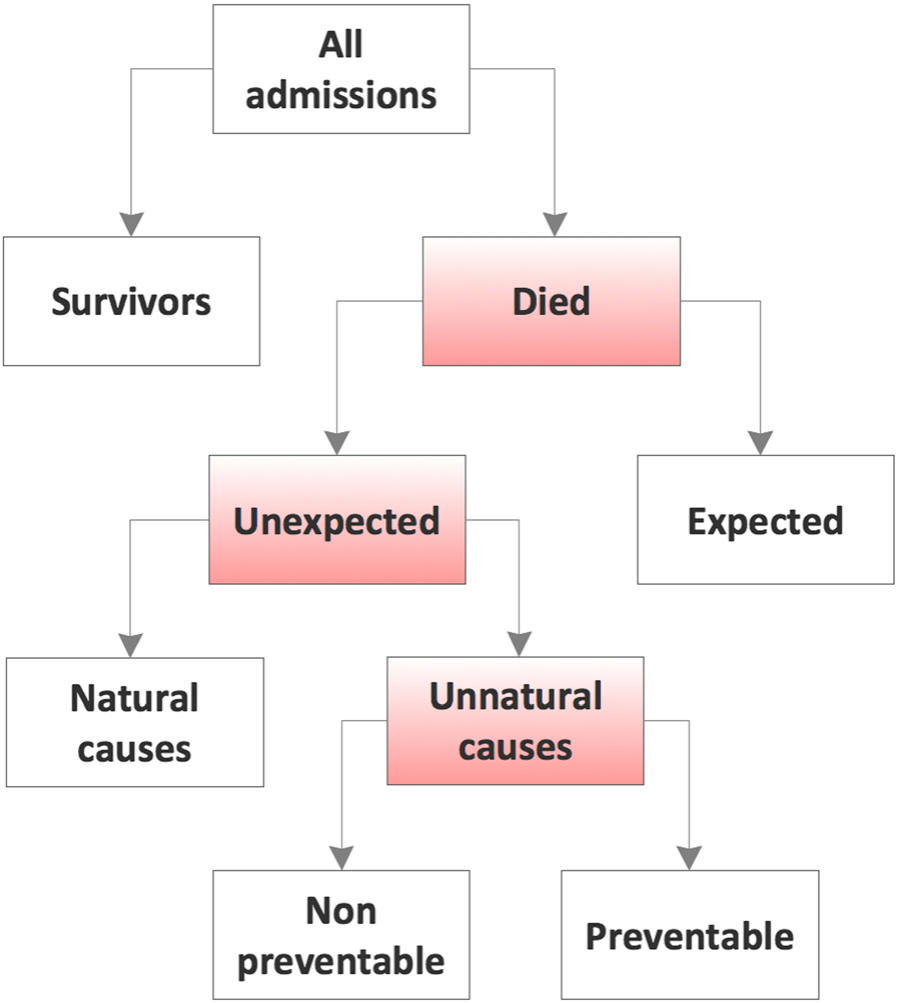
Taxonomy regarding all hospital deaths according to Norwegian regulations

For the first part of the hospital record screening, four investigators (HF, RSA, BAA and JHR) independently screened records of hospital deaths and initially classified the death as unexpected or not unexpected (Fig. 1). It is mandatory to document this in official Norwegian death certificates where a box have to be ticked if the death is considered unexpected and/or with sudden onset. No formal training for this task was done, but two of the physicians had extensive training in journal screening through the hospital adverse event reporting system (hospital auditory committee). Disagreements at this stage were discussed until a consensus was reached. If not resolved, the death was classified as an unexpected death. When the death was classified as sudden or unexpected, it was obvious from the medical record that the patient was not in the final stage of a severe, chronic, or terminal disease and that death was forthcoming. The remaining deaths were classified as expected, although no strict criteria were given for this group of deaths as they are considered more of an exclusion group for not being sudden or unexpected according to Norwegian law. In the end of this stage, when a death was considered sudden and unexpected, unnatural causes of death must be accounted for, if present, using a list of specified conditions defined according to Norwegian law: § 2 in “Regulation of physicians’ report to the police about unnatural deaths” 2001 (Table 1), in particular section E.

In part two, all deaths classified as unnatural were discussed by a reference group of six consultants from surgery, anaesthesiology and emergency medicine, intensive care, internal medicine, pathology, and the hospital patient safety unit during a one-day meeting. No formal training for this group were done, but we considered it important that this group represented a broad clinical experience since some of these deaths are often extremely difficult to judge in particular with regard to preventability. Consensus about the causes of unnatural deaths was sought. In addition, signs of any adverse event during the hospital stay were discussed and a consensus was reached as to whether or not (≥50% likelihood) the death could have been prevented. To answer the latter question, a five-point Likert scale was used: 1 was scored as not preventable, 2 as possibly not preventable, 3 as uncertain preventability, 4 as possibly preventable, and 5 as preventable.

MedCalc v 12.7.00 for Windows (MedCalc Software) was used for comparisons between groups and calculation of 95% confidence intervals (CI) for mean and median values. Kaplan Meier curves were produced using JMP software (SAS Institute Inc).

### Ethical approval

The study protocol was approved by the Regional Research Ethics Committee of the Western Health Region (2012/564). They judged the study to be a quality assurance project of existing hospital data and therefore waived informed consent. The project was also approved by the data protection officer (personvernombudet) Bergen Health Trust (2015/566). After extraction of clinical data from the patients’ files and analysis of each death, all further analysis and group discussions were performed using de-identifiable data.

## Results

**Fig. 2:**
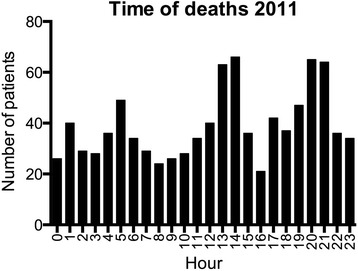
Frequency of deaths according to the hour of the day

Most of the recorded 1185 deaths (1.98% of all admissions) occurred in typical “medical departments” such as the Cardiac, General Medical, or Pulmonary Departments (Table 2). The median and mean ages of all patients who died were 78 and 73, 8 years, respectively. There were no significant differences in deaths throughout the year, but the time of death during the day peaked around noon (13–14 h) and in the evening (20–21 h) (Fig. 2). The median time from admission to death was 5.6 days.

**Table 2:** Characteristics of patients who died in the hospital

Group	N	Age (years) Mean and (median; 95% CI)	CCI Mean (95% CI)	LOS (days) Mean	Died in ICU (%)
All	1185	73.8 (78)	6.9	8.4	11.6
Expected	895	74.5 (78)	7.4 (7.2–7.6)	9.1	8.7
Unexpected	290	71.5 (78)	5.5 (5.1–5.8)	8.2	20.3
Natural	218	73.5 (79; 77–81)	5.7 (5.3–6.0)	8.4	18.8
Unnatural	72	66.0 (69; 64–76)	4.8 (4.0–5.5)	7.6	25.7

Patients deaths were classified as expected or unexpected, and as due to natural or unnatural causes. *CI* confidence interval; *CCI* Charlson Comorbidity Index; *LOS* length of stay; *ICU* intensive care unit

**Fig. 3:**
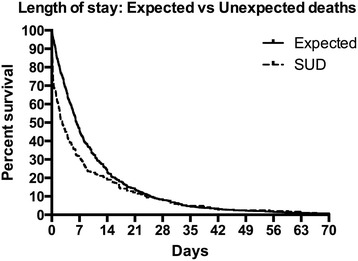
In-hospital survival prior to death in patients whose death was classified as expected versus unexpected

We found 290 (24.5%), unexpected deaths leaving 895 deaths classified as expected (75.5%). Of the 290 unexpected deaths, 72 (6.0% of all deaths) were classified as unnatural deaths (Table 3) and the remaining were considered to be due to natural causes. In 16 deaths (1.4%), we identified evidence of adverse events or factors that could have been prevented or may have contributed to the death (Table 4). In 18 deaths (1.5%), it was not possible to determine preventability. This implies that in 34 deaths (2.9%) there was ≥50% likelihood that death was preventable. Both hospital LOS and CCI were higher among patients in the group of expected deaths, and the lowest values were found in the group of unexpected unnatural deaths. The Kaplan-Meier curves of hospital stay for the groups of expected or sudden and unexpected are shown in Fig. 3.

**Table 3:** Differences in number of deaths according to clinical units

Units	Number	Age (mean)	LOS mean	SUD (%)#	Un-nat (%)#
Surgical	235	74.5	8.4	90 (31)	28 (40)
Medical	349	78.9	8.5	77 (27)	11 (16)
Pulmonary	150	74.3	8.8	20 (7)	4 (6)
Cardiology	183	73.9	5.2	74 (25)	18 (26)
Miscellaneous	268	66.2	9.6	29 (10)	9 (13)
All units	1185	73.8	8.4	290	70

*LOS* Length of stay, days, *CCI* Charlson Comorbidity Index, *SUD* Sudden unexpected death, *Un-nat* unnatural deaths according to Norwegian law. # % of number of deaths in the group

In text: The highest proportion of deaths classified as sudden unexpected deaths and unnatural deaths was found in the Department of Neurosurgery (49 and 26% respectively)

**Table 4:** Possible preventable events leading to death

Age	Event(s) leading to death
85	Pneumonia, ultrasound guided biopsy. Not monitored. Next night developed signs of septic shock with hypotension, lactate 18 mmol/l and severe hypoxemia. Died. Death probably related to the biopsy.
90	Isoprenaline infusion with a syringe pump. The hosing lost connection with syringe, and before this was detected the patient development of therapy resistant bradycardia and death.
60	Admission with suspected endocarditis, not monitored. Had cardiac arrest on ward, resuscitation efforts negative. Died.
64	Elective surgery for liver metastasis. Perioperative lesion of the liver vein with profuse bleeding. Death on the operating table.
73	Admitted with tentative diagnosis: urethral stone, and was treated for this. Patient suddenly developed circulatory arrest and died. Post mortem autopsy revealed peritonitis and perforated colon. Error of omission.
80	Whiple’s operation performed. In recovery room delirious, and a new gastric tube had to be reinserted. This resulted in vomiting and pulmonary aspiration leading to cardiac arrest and death.
77	Urethral catheter inserted which resulted in profound urethral bleeding and hypovolemic shock. Next day severe sepsis secondary to urinary tract infection. Death.
57	Iatrogenic opiate overdose postoperatively. Found dead in bed. Probably related to opioid overdose.
68	Thoracic drain inserted to remove pleural effusion. After several hours development of circulatory shock and anemia. Died. Post mortem exam revealed large amount of blood in thoracic cage.
64	Postoperative pneumothorax during mechanical ventilation. Insertion of pleural drain resulted in bleeding from an intercostal artery, leading to thoracotomy because of ongoing bleeding. Had a cardiac arrest. ROSC, but severe cerebral injury led to withdrawal of treatment some days later.
80	Pleural drain inserted. Resulted in bleeding and cardiac arrest. Received anticoagulation drugs.
66	Cancer pulm. Operated. After surgery airway problems (ET tube) with hypoxemia and hypotension. Did not wake up, and treatment was stopped after 6 days.
60	Abdominal pain, given ketobemidon. Low body weight. Registered low respiratory rate during next night, nothing was done and patient found dead in the morning. Possible opioid overdose.
81	Because of delirum given klometiazol (Heminevrin) i.v. One hour later cardiac arrest and with no ROSC. Died.
89	17 days in hospital with abdominal pain, no diagnosis made. Patient died. Post mortem revealed gallstone and cholecystitis. Error of omission
86	Dyspnoe and AMI, given antithrombotic drugs that resulted in profound bleeding and haemorrhagic shock. Death.

There were differences in the proportion of sudden and unexpected deaths and also unnatural deaths according to type of clinical department (Table 2). Departments of Surgery, Medicine and Cardiology had the highest proportion of their deaths classified as sudden, unexpected and un-natural. The lowest proportions were found in a mixed group of Departments, with considerably fewer deaths, as Ophthalmology. ENT, Neurology, Gynaecology and Obstetric to mention some. The single unit with the highest proportion was found at the Department of Neurosurgery with 49% sudden or unexpected deaths, and 26% considered as un-natural.

## Discussion

In this study, we found that approximately 2% of all hospital admissions ended with the patient dying during their hospital stay. The majority of deaths occurred in ordinary hospital wards, not in the intensive care units or intervention/operating theatres. Approximately one out of four hospital deaths was considered to be unexpected and/or sudden. Only a minor proportion of deaths (6%) were classified as unnatural, and 2.9% were considered to have ≥50% likelihood of being preventable.

There are very few published papers about death in Norwegian hospitals in general. We know from Statistics Norway that 41.304 citizens in Norway died in 2011, and that 14,452 (34.9%) of these deaths occurred in hospitals [6]. Hence, 1189 (8.2%) of all Norwegian in-hospital deaths occurred in our health trust, which serves approximately 8% of the population. In a previous study of 496 deaths from a single hospital in Norway in 2008, the quality of the death certificates was studied prospectively [7]. Incorrect information was found in 20% of all death certificates, and 12% of all deaths were due to unnatural causes; lethal adverse drug reactions comprised 5% of deaths. Interestingly, a retrospective analysis of deaths from two previous months that were presented as controls showed that the proportion of unnatural deaths was 7%, a figure not very different from the 6% found in our retrospective study. This may indicate that a prospective analysis at the time of death is a better approach for exploring the circumstances around unnatural deaths than a retrospective one.

In general, two different methods are used to identify unnatural causes of death in hospitals. The first is a traditional method that analyses the patients that die in either a representative sample or all deaths during a certain period. The other is to study a group of patients exposed to an adverse event and follow their outcome. Adverse events can be studied in several ways, from using specific reporting systems to retrospectively screening patient files. The latter method has become very popular since its first use in the 1990s and has recently been systematised through the Global Trigger Tool (GTT) method [8]. In this method, a random sample of patient hospital files is screened at regular intervals to determine the magnitude and consequences of adverse events.

The GTT method was introduced in Norway in 2011 and is now routinely used in many Norwegian hospitals [9].

In a recent study from Portugal, the adverse event screening method was used to estimate the incidence of adverse events in hospitals and their relevance to hospital deaths [10]. A random sample of 1669 medical records from three acute care hospitals from 2009 was analysed using a structured record review based on 18 criteria. The study found an 11.1% incidence of adverse events, with approximately 50% considered preventable. Of these, 10.8% were associated with deaths. This means that approximately 1.2% (20) of their random sample (12 out of 1000 patients admitted to hospital) died from an adverse event.

The number of deaths in Norwegian hospitals has also been estimated using data from the GTT campaign [11]. This project found similar incidences as in Portugal; i.e., adverse events contributed to a high proportion (0.66%) of all hospital admissions leading to death in 2010. Transformed to all hospital admissions in our country in 2010, this equates to 4500 deaths or 34% of all hospital deaths.

This way of estimating hospital deaths seems regularly to give higher numbers of patients dying from adverse events than when only the population that actually dies in the hospital is specifically investigated. There might be several explanations for this discrepancy. When using the GTT to investigate random hospital admissions, only a small proportion of patients reviewed will die during their hospital stay. Also, the variation from sample to sample will most likely be large as well as the margin of error. Such random sample estimations should therefore regularly be compared to a review of all deaths in a hospital or region with specific regards to the frequency of adverse events leading to death.

Studies using our methods are infrequently published from Europe, but have recently been presented from the UK and the Netherlands. In the Netherlands [4], a large study from 2008 on adverse events including a large sample of hospital deaths found evidence of adverse events in 10.7% of all patients who had died. Of these, 4.1% of the deaths were deemed preventable. In a recent follow up, deaths due to adverse events in Dutch hospitals during 2011–2012 were reported to be further reduced to 2.6% [12]. This is a time-period similar to our study with comparable results on preventable deaths of 2.9%. In a UK [3] study of 1000 hospital deaths that occurred in 10 acute care hospitals, reviewers judged that 5.2% of the deaths had a ≥ 50% chance of being preventable.

The first application of GTT from 2010 to 2011 in five Danish hospitals was recently published [13]. Interestingly, and in contrast to the Norwegian safety campaign study, none of these events was found to lead to a fatal outcome.

The link between detection of an adverse event and its consequence is also difficult to determine. Very often, the observed consequence (effect) of an event is mixed with the adverse event, making it difficult to separate the two. As an example, if the adverse event is a miscalculation in the given dose of a particular drug, the consequences could range from no event (most likely) to death (very infrequent), but the initial adverse event remains the same. In particular, the link between adverse events in patients who later expire in the hospital is challenging to identify. The expertise of the screening teams may not always be sufficient to evaluate such events in adequate depth since competence and insight into the particular medical field is important.

Several of our unnatural deaths occurred in the operating theatre in patients who were critically ill or who had near-lethal conditions prior to hospitalisation (e.g. multiple trauma or cardiac arrest). Critically reviewing such deaths is extremely difficult in the context of adverse events given the complexity of such situations.

We found that the majority of the deaths in our hospital were expected according to circumstances present at admittance or that became obvious during the patients’ hospital stay. Since we do not have a comprehensive hospice system, it is also common for Norwegian hospitals to admit patients whose death is imminent in order to provide symptom relief, comfort, and care until death. In this particular group of patients, we argue that the use of our traditional understanding of adverse events could be of limited clinical value. Very often, the final common path to death from a terminal illness is a respiratory “complication”, such as aspiration or pneumonia, leading to respiratory failure and, in the end, cardiac or respiratory arrest. Such conditions, although possibly preventable, do not conform to the concept of an adverse event that we would necessarily aim to correct or prevent. Perhaps errors of omission, such as inadequate pain and symptom control, are more important in this group of patients. However, we did not have sufficient data to analyse this. This is why the first part of our study was to document whether or not a death was expected and, if the cause of death was probable from the disease process, this was categorised as an expected death. In our opinion, it is mainly in the group of unexpected deaths that the term “lethal adverse event” is clinically meaningful.

As with all retrospective analyses, our study has several limitations. Necessary and detailed information about the occurrence of adverse events is not always documented in the patient files. This is particularly true for errors of omission. We did not follow a strict procedure regarding our journal review of deceased patients, and we did not calculate the inter-observer variations. Keeping our study to the method of classifying deaths in Norway is probably also different from clinical practice in other countries. The judgement of the “expert” panel is also context specific, and we could have had too strict of a definition regarding unexpected deaths as a group. We may also have been too strict to judge adverse events as non-preventable. All of the participants on the panel were from Haukeland University Hospital. Hence, we may have been less critical of our own hospital’s performance than if we had employed an external review group. Lethal adverse events may also occur in patients with life threatening conditions or, e.g., were in a final stage of cancer. Therefore, our estimate may be somewhat lower than the true situation; however, we do not consider this a major bias.

Based on the results from our study, we recommend caution when analysing and drawing conclusions on the cause of death using the random sample method of adverse events without considering what type of patients are being studied. In particular, using such small sample sizes to estimate the national burden of deaths, as was initially done by the Norwegian National Safety Campaign, could be biased. If the primary aim is to estimate the proportion of adverse events contributing to death, we recommend also specifically studying the total population of patients who actually died in the hospital and, thereafter, analysing the causes of death in that population.

## Conclusions

We found that approximately 2% of all hospital admissions in our health trust died after admission. The majority of deaths occurred in ordinary hospital wards. Approximately one out of four hospital deaths was considered to be unexpected and/or sudden. Of all deaths, only a minor proportion (6%) were classified as unnatural, and half of these were considered to have ≥50% likelihood of being preventable.

## Abbreviations

CCI: Charlson Comorbidity Index
CI: Confidence interval
GTT: Global trigger tool
ICU: Intensive care unit
LOS: Length of stay
SUD: Sudden unexpected death
